# Not exodus, but population increase and gene flow restoration in Cantabrian brown bear (*Ursus arctos*) subpopulations. Comment on Gregório et al. 2020

**DOI:** 10.1371/journal.pone.0240698

**Published:** 2020-11-02

**Authors:** Juan Carlos Blanco, Fernando Ballesteros, Guillermo Palomero, José Vicente López-Bao

**Affiliations:** 1 Fundación Oso Pardo, Santander, Spain; 2 Research Unit of Biodiversity (UO/CSIC/PA), Oviedo University, Mieres, Spain; University of Warsaw, POLAND

## Abstract

In a genetic study on brown bears (*Ursus arctos*) in the Cantabrian Mountains, Gregório et al. (2020) interpreted the asymmetrical gene flow they found from the eastern subpopulation towards the western one as an exodus of bears forced to flee from the eastern nucleus “with higher human disturbance and poaching”, concluding that connectivity may be operating as a means for eastern Cantabrian bears to find more suitable territories. In this reply, we maintain that the explanations of Gregorio et al. contradict the source-sink theory and we also present demographic data not considered by these authors showing that the eastern subpopulation is not declining, but persistently increasing. After reviewing the demographic and genetic studies published during the last 20 years, we conclude that the connectivity between the two subpopulations is operating as a route which allows the regular movement of males and the restoration of the gene flow across the whole Cantabrian population.

## Introduction

During recent decades many populations of large carnivores have been recovering [1] and it is important to properly describe the features of the recovery processes to plan the adaptive management [2]. The endangered population of brown bears (*Ursus arctos*) in the Cantabrian Mountains (northwestern Spain) was on the verge of extinction in the late 1980s and early 1990s, but following the implementation of conservation measures it is now increasing in numbers and expanding in range [3].

In a recent genetic study on this population of brown bears, Gregório et al. [4] confirmed that it is structured in two subpopulations (eastern and western), as previously described [3, 5–9]. These authors also present more evidence of male mediated bidirectional gene flow and admixture between both subpopulations [3, 8]. However, Gregório et al. [4] found an asymmetrical and more intense gene flow from the smaller eastern subpopulation towards the larger western subpopulation, raising the intriguing question that headed the title of their article: does the recent reconnection of both subpopulations mean a process of colonization or of an exodus of bears from the eastern subpopulation to the western one? The authors tended towards the exodus option, and proposed (p. 15) that “bears could be forced to flee from areas with higher human disturbance and poaching, which are more intense in the eastern Cantabrian Mountains”; “It is reasonable to infer that individuals from the eastern Cantabrian subpopulation might be dispersing westwards to seek habitats with less human interference and to escape human persecution”; “Thus, instead of promoting colonization (and reinforcement) of the eastern Cantabrian region by bears from the western Cantabrian Mountains, connectivity between the two subpopulations may operate as a means for eastern Cantabrian bears to find more suitable territories.”

The article by Gregório et al. [4] makes an interesting assessment of the recovery process of this bear population from the genetic point of view, but in our opinion it fails when making an ecological interpretation of the data. Furthermore, their interpretation suffers in our opinion from a major flaw, in that they have not used well-known data on the demographic trend of the eastern bear subpopulation.

In this article we want to detail the information on the demography of the Cantabrian bear population (especially of the eastern nucleus) and interpret the available genetic and demographic information from the perspective provided by previous studies undertaken on bears in the Cantabrian Mountains and in the light of the currently accepted ecological framework. Our goal is to offer scientists and managers working on this and other endangered bear populations an alternative perspective regarding the continuing recovery of bears in the Cantabrian Mountains.

## Methods

To answer the question posed by Gregório et al. [4] (“Paths for colonization or exodus?”) we use four approaches. Firstly, we discuss the exodus concept *vs*. the source-sink theory applied to brown bear ecology and the situation of the Cantabrian brown bear population. Secondly, we review the available evidence to assess whether human disturbance and persecution (illegal hunting) of brown bears can result in a massive migration of individuals (exodus). Thirdly, we offer an alternative interpretation considering the available genetic information on the Cantabrian bear population published to date. Finally, we provide crucial demographic information for this bear population that is missing in the paper of Gregório et al. [4].

### Monitoring females with cubs and mortality

The main source of data on the demographic trend of bears in the Cantabrian Mountains comes from monitoring groups of females with cubs. The annual survey of females with cubs of the year started in 1989, is used as a proxy of the population trend [3, 10–12] and has been carried out continuously until now by a coordinated team of technicians and rangers from four regional administrations and the NGO Brown Bear Foundation, with the collaboration of other NGOs, research institutions and rangers. The method has been described several times [3, 10, 11] and this survey is particularly detailed in the eastern subpopulation because it is much smaller than the western one and the relative effort devoted per female is higher.

The figures on mortality come from a data base kept by the Brown Bear Foundation since 1990 which includes information on all the bears found dead and the official necropsies carried out by the Veterinary College of Leon University and other institutions.

## Results and discussion

### Exodus vs. source-sink theory

The Lexico Oxford dictionary (https://www.lexico.com/definition/exodus) defines exodus as a mass departure of people (e.g. *the annual exodus of sun-seeking Canadians to Florida)*. Exodus is also the Bible text that narrates the slavery of the Hebrews in ancient Egypt and their liberation through Moses, who led them to the Promised Land. In Gregório et al. [4], exodus evokes a massive movement of eastern bears escaping from the presumed persecution in this area, in their search for the much better conditions experienced by the western subpopulation.

Nevertheless, in ecology, the dynamics of spatially structured populations (like that of Cantabrian brown bears) is usually explained in the framework of the source-sink theory [13–15]. According to most studies, human-caused mortality produces home range takeover in carnivores. Dispersal of individuals from low to high mortality areas (compensatory immigration) has been documented for different carnivore species, in which unevenly distributed hunting pressures induce source–sink dynamics [16–19]. In the case of bears, Lamb et al. [20], using 41 years of demographic data for more than 2,500 brown bears in the Rocky Mountains, concluded in a recent publication that population growth rates for bears in human dominated areas revealed a source-sink dynamic. Despite some female bears successfully reproducing in areas with a high human caused mortality (the sink areas), bear persistence was reliant on a supply of immigrants from areas with minimal human influence (the source areas). This source-sink dynamics (migration from low to high human-caused mortality areas) had previously been reported by other important works on brown/grizzly bears, both in North America [21–24] and in Europe [25–28].

Brown bears have been continuously present in the eastern Cantabrian Mountains [29], and a recent habitat assessment has shown that habitat characteristics are similar in both Cantabrian subpopulations [30]. Under these circumstances, the removal of individuals will produce an attractive sink [15], as has been specifically suggested for this bear population [31]. In consequence, according to the source-sink body of evidence, the alleged removal of bears in the eastern subpopulation through poaching would not cause an extensive migration or “exodus” of bears to the western subpopulation, but the opposite: the migration of bears from the western to the eastern subpopulation in order to fill the gaps. In that case, the eastern subpopulation would become a sink and not a source as Gregório et al. [4] suggest in their paper. We are not aware of any publication that describes the mass dispersal (exodus) of large carnivores from areas with high mortality to areas with low mortality.

### Would human disturbance or persecution cause an exodus of bears?

We will assess the aspects related to bear mortality in both subpopulations in the last section, but it is unlikely that human disturbance is higher in the eastern subpopulation since human population density is lower here (7.1 inhabitants/km^2^) than in the western one (11.0) [32]. Tourism also seems to be also higher in the western subpopulation since Somiedo Natural Park alone presumably attracts more tourists (130,000 in 2018 [33]) than the sum of protected areas of the eastern subpopulation (e.g., 20,474 tourists in 2018 in Fuentes Carrionas Natural Park [34]). In addition, the human footprint (i.e. landscape variables associated with humans) of the habitat is also higher in the western subpopulation [30].

We have reviewed the scientific literature to assess if human disturbance or poaching (bears are strictly protected in Spain) could cause an exodus from the eastern subpopulation in search for a quieter and safer habitat in the western one, as suggested by Gregório et al. [4]. To cope with these issues they use several strategies. Bears mainly shift their activity period to become more nocturnal. Ecotourism on Alaskan salmon streams displaces large male bears in time rather than in space–during the viewing day large males were less active than at other times [35]. Brown bears in Alberta, Canada, moved away from human development in periods of high human activity (0700–1800 hours) and were closer when human activity diminished (1800–0700 hours) [36]. Movement patterns of brown bears in Scandinavia were studied before and after the start of the annual bear hunting season. Solitary bears subject to hunting increased their movements during the hours of darkness after hunting started, losing their nocturnal rest probably to compensate for decreased daytime activity. Females with cubs-of-the-year, which are protected from hunting, also modified their movement pattern, but much less than hunted bears [37]. Brown bears living near people in the Rocky Mountains shifted to a nocturnal activity pattern as they aged, increasing their nocturnality by 2 to 3% per year past the age of 3, which led to a 2 to 3% increase in survival per year; this shift was not detected in wilderness areas [20].

In addition to activity shifting, bears show other adaptations to avoid humans. In Sweden, in summer/fall, with more intensive and dispersed human activity, including hunting, brown bears rested further from human settlements during the day and beds were better concealed in dense cover than in spring. Bears adjusted their behaviour to avoid human encounters and responded to fine-scale variations in human-derived risk, both on a seasonal and a daily basis, but they did not abandon the area [38].

In addition, a number of experiments have been carried out in Sweden to assess the behaviour of radio-collared brown bears disturbed by researchers who mimicked hikers (or hunters) approaching them on foot. Solitary bears disturbed by people on foot become active for 24±23 min (n  =  78), and moved on average 1,173±1,094 m (n  =  92) before they settled at the second site [39]. Females with cubs moved greater distances (1,200–2,300 m) than solitary bears and spent more time active following the approach. After disturbance, they settled into denser habitats [40]. When they were approached by people with dogs (as Scandinavian bear hunters do), bears moved the largest distance (3,482 m) before settling [41]. The immediate reaction after the experimental encounters caused an average 26% increase in distance travelled by the bears compared to the same time of the day during the week prior to the approaches, immediately followed by a 10% reduction in movement. Bears moved more during the darkest part of the 2 nights following an approach and they showed a reduction in daytime movements in the 2 days following the encounters [42]. Nevertheless, none of the experimentally disturbed bears left the area where they were living.

In conclusion, although the effects of hunting and human disturbance on brown/grizzly bears have been studied in great detail both in America and Europe, we have no detected any publication reporting an exodus (massive long distance migration) of individuals as such proposed by Gregório et al. [4].

### The migration of males between both Cantabrian subpopulations: A temporal perspective

Previous to Gregório et al. [4], the demography and genetics of the Cantabrian bear population had been the object of a number of studies, whose proper review can give us valuable insights. In summary, these studies have shown 1) that the Cantabrian bear population is dynamic and has been increasing over the last 25 years, as stated by several authors using surveys of females with cubs of the year [10, 11], genetic surveys [9] and a combination of both methods [3]; 2) that the Cantabrian population is genetically structured in two, western and eastern, subpopulations [3, 5–9]; 3) that there is migration of males (but not females) between both subpopulations, which has increased as the Cantabrian bear population has increased. This migration was first detected in 1992, when a male with a genetic profile of the western subpopulation was identified on the eastern side [5]. Subsequent migration activity was detected in 2004–2007 with west-east movements of three males and an east-west movement of one male [8]. Gene flow between the subpopulations was first detected in 2008 based on two genetically admixed individuals sampled in the eastern subpopulation [8]. Finally, a study genotyped 116 samples from the eastern and 36 from the western subpopulations collected in 2013–2014 [3]. Of the 26 unique genotypes detected in the eastern subpopulation, 14 (54%) presented an admixture composition, and seven (27%) were determined to be migrants from the western subpopulation.

Gregório et al. [4], with 142 samples collected mainly (86%) in 2016 and 2017, detected bidirectional gene flow and admixture among the subpopulations, but in their study gene flow was significantly more intense from the eastern to the western Cantabrian subpopulations (they only detected first generation male migrants from the eastern to the western Cantabrian subpopulation). But even in this study, 20% of the unique bear genotypes of the eastern subpopulation (6/30, Fig 3A in Gregório et al. [4]) were admixed individuals, which shows a significant gene flow from the western to the eastern subpopulation prior to 2016–2017. In fact, the proportion of migrant plus admixed males in the western subpopulation (26%: 13/50, Fig 3A) is just a bit higher than the percentage of admixes in the eastern one (20%). Admixed individuals are more relevant than migrants to detect effective migration and gene flow, because there is not always a direct relationship between demographic and genetic connectivity [43]. Pulling together the evidence collected by different studies over the last three decades, the most plausible interpretation is that males started to move between both subpopulations when the Cantabrian population started to increase, in the 1990s [3], and this movement has been increasing until today, with an apparently regular flow of males occurring between both subpopulations. Since dispersal in brown bears is sex biased, this regular migration of males (but not females) in the Cantabrian Mountains matches with the general pattern described in other brown bear populations [44–46] rather than with an exodus, which should also involve female bears, equally affected by human disturbance.

Unlike Gregório et al. [4], all previous studies have found a predominant flow of males from the western to the eastern subpopulation [3, 5, 8], an asymmetric gene flow similar to that found in other populations [47]. The differences in the direction of the gene flow detected in different studies may be related to the limited number of samples collected by these studies or because change in dispersal magnitude and direction is an inherent property of a dynamic recovering metapopulation [48]. For example, Gonzalez et al. [3] did not detect eastern migrants in the western subpopulation maybe because they only collected 36 samples from this nucleus. In any case, the review of the genetic studies published since 2000 clearly show that the movement of males between both subpopulations is bidirectional.

The gene flow of males into the eastern subpopulation and the consequent admixture from 2008 onwards [8] has apparently produced an improvement of the genetic condition of this subpopulation, which has been reflected in an increase of the genetic diversity [3, 4] and of the occurrence of litters with three cubs. For example, in the 19-year period 1989–2007, only one female with 3 cubs was detected (in 2005) in the eastern subpopulation, while in the 12-year period 2008–2019, 6 reproductive events with three cubs were detected (in 2012, 2014, 2016, 2018 and two in 2019).

### Population trend in the Eastern subpopulation

Gregório et al. [4] claim that the eastern subpopulation is declining: “Among other causes of population decline, it is possible that the Eastern Cantabrian subpopulation is actually “losing” individuals to the Western Cantabrian subpopulation.” (p. 15). However, this statement frontally contradicts the demographic surveys of females with cubs of the year (hereafter FCOY) carried out in the Cantabrian Mountains. This information on females is particularly useful to complement the genetic data obtained by Gregório et al. [4], which are male-biased: 25 of the 30 unique genotypes captured in the eastern subpopulation corresponded to males and only 5 to females (S4 Table). Previous genetic studies in the Cantabrian Mountains found that “the male to female ratio of the sexed individuals was 1.14:1. Given that females have a lower probability of capture than males, the observed sex ratio is probably skewed towards females” [9].

**Fig 1:**
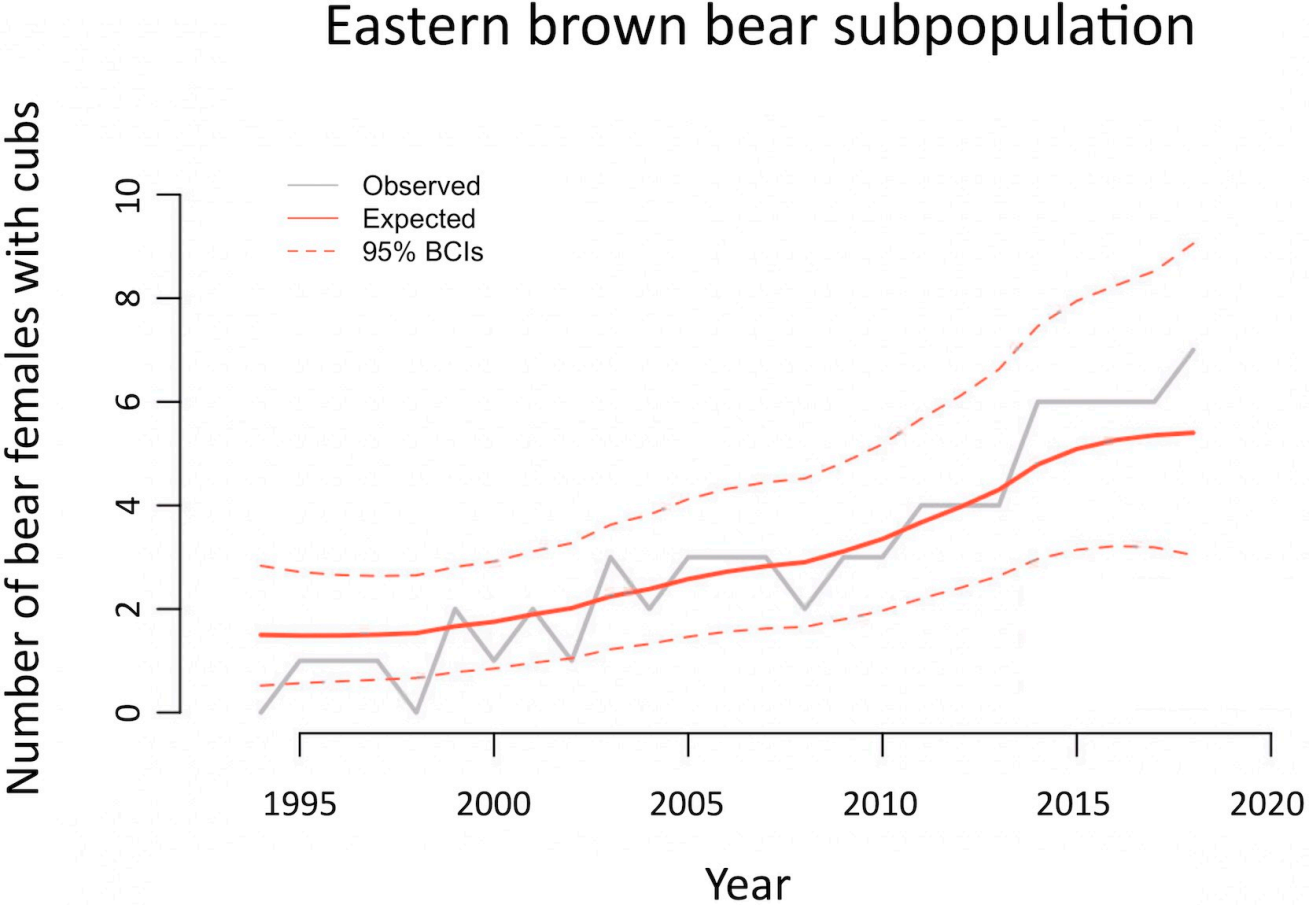
Trend in the number of females with cubs of the year (FCOY) detected annually in the Eastern Cantabrian subpopulation between 1994 and 2018 (grey solid line–observed). Population change over time (red solid line–Expected) was estimated using a Bayesian time-series modelling approach using the R-INLA package for R [54]. We used a Negative Binomial error distribution and included an auto-regressive term, since the number of FCOY detected annually are not independent over time.

The annual FCOY survey in the eastern subpopulation shows a slow but continuous increase since the mid-1990s (Fig 1). The eastern subpopulation went from 1 FCOY detected in the biennium 1994–95 to 13 in the biennium 2017–18, and since 2009 the number of FCOY detected in a given year equals or outnumbers those of any previous year. A recent study [3] showed that the rate of exponential growth from 1994 to 2014 was almost equal for the western (10.1%) and the eastern (10.4%) subpopulations. In the period 1994–2018, this figure is still 10.0% for the eastern subpopulation (95% Bayesian Credible Interval: 6.0–14.0%; Poisson regression using the package “brms”: [49]) (Fig 1).

Considering that the average litter interval for female brown bears in the Cantabrian Mountains is 2.2 years [50], the latest available figures of FCOY (6 in 2017 and 7 in 2018, Fig 1) indicate that there are at least 14 reproductive females in the eastern subpopulation, a much higher figure than the ~4 reproductive females that Gregório et al. [4] (p. 15) claim for this subpopulation. The number of ~4 reproductive females is also inconsistent with the 25 unique genotypes of males captured by Gregório et al. [4] in the eastern population, even accepting that this number must not be considered as a census.

The scarce genetic population estimates of the eastern subpopulation are consistent with the demographic trends. In autumn 2017, a study using both microsatellites and SPNs estimated 48.6–52.7 bears in the eastern nucleus [51], which represents an obvious increase over the 19 bears estimated by a previous genetic population estimate in 2006 [9]. In addition, the estimates of 2017 are consistent with the surveys of FCOY, since a healthy brown bear population is composed of 8–12% females with cubs of the year [51–53].

Gregório et al. [4] presume that bears from the eastern subpopulation “could be forced to flee from areas with higher human disturbance and poaching, which are more intense in the Eastern Cantabrian Mountains”, but there is no evidence supporting this statement beyond the suspicions suggested by Lamamy et al. in the Discussion of their paper [30]. According to the information of our data base, from January 2000 to August 2020, 8 bears have been found illegally killed (verified by official necropsies) in the western subpopulation (3 poisoned, 3 snared and 2 shot) and 4 (3 poisoned and 1 shot) in the eastern one. It is difficult to obtain conclusions from these opportunistically collected data, since the search effort, the accessibility of the area and other non-quantifiable factors may affect the probability to find bear carcasses. Although poaching exists in both Cantabrian nuclei, in recent times it has not prevented an increase in bear numbers in a similar rate for both subpopulations.

## Conclusion

Contrary to the statement of Gregório et al. [4], the available information does not support a decline in the eastern Cantabrian bear subpopulation, but a slow and sustained increase, which is obvious both from the annual surveys of FCOY and from the genetic population estimates. This increase discredits the hypothesis of an exodus of bears from the eastern to the western subpopulation. The connectivity between the two subpopulations is not operating as a means for eastern Cantabrian bears to escape human persecution and find more suitable territories, as Gregório et al. [4] claim, but as a route which allows the regular movement of males across the whole Cantabrian population and the restoration of the gene flow both in the western and eastern subpopulations.

However, we fully agree with Gregório et al. [4] that more efficient control over bear poaching and greater efforts to raise public awareness in order to reduce human-bear conflicts are important tasks to secure the conservation of the Cantabrian brown bear population.
